# Seroprovalence of herpes simplex1, 2 IgG antibodies in patients with beta thalassemia in a major tertiary care hospital located in Yazd, Iran

**Published:** 2014-04-20

**Authors:** A Atefi, F Binesh, A Hashemi, A Atefi, MM Aminorroaya

**Affiliations:** 1 MSc Of Microbial Biotechnology, Shahid Sadoughi University Of Medical Sciences,Yazd,Iran; 2 Department Of Pathology, Shahid Sadoughi University Of Medical Sciences,Yazd,Iran; 3 Department Of Pediatric Medicine, Shahid Sadoughi University Of Medical Sciences,Yazd,Iran; 4 BSc Of Nursing, Shahid Sadoughi Hospital,Yazd,Iran; 5 Clinical laboratory, Shahid Sadoughi Hospital,Yazd,Iran

**Keywords:** Thalassemia, Seroprevalence, Human herpes simplex virus

## Abstract

**Background:**

Patients with beta thalassemia suffer from increased susceptibility to infections and putridity plays a major role in the patient's morbidity and mortality. The risk of transfusion-transmitted viral infection is well known in these patients. However, there is dearth of information about the seroprevalence of herpes simplex virus (HSV) infection in patients with beta thalassemia in literature. This study analyzes the prevalence of anti-HSV1, 2 IgG antibodies in patients with beta thalassemia in a major tertiary care hospital located in Yazd,Iran.

**Material and methods:**

In this case control study, we undertook a serological study of HSV1,2 IgG antibodies among 45 patients with beta thalassemia and 45 healthy individuals as control group by ELISA method. A p.value <0.05 was considered statistically significant. Statistical analyses were performed using SPSS.20.

**Results:**

The prevalence of HSV 1,2 IgG antibodies were estimated 88.8% among patients with beta thalassemia and 77.7% in control group. Regarding p.value=0.64, it showed no significant difference in these two groups.

**Conclusion:**

Although infectious diseases still represent a major challenge in patients with beta thalassemia, HSV past infection rate was not increased in these patients in our study. More studies are required to clarify this matter.

## Introduction

The thalassemias are inherited disorders caused by mutations that decrease the synthesis of α- or β-globin chains. As a result, there is a deficiency of hemoglobin and additional red cell changes due to the relative excess of the unaffected globin chain (1). Thalassemia is considered the most common genetic disorder worldwide. According to data collected through the Hereditary Disease Program of the World Health Organization and based on local surveys and reports by visiting experts, the carriers of hemoglobin disorders in the world are estimated to be 269 million(2). Iran is located in the middle of the thalassemia belt (3). After heart failure, infection is a major complication and the leading cause of death in patients with beta thalassemia. Prompt recognition with timely treatment is essential in the treatment of these patients. In other word, these patients have tendency to infect with many bacterial and viral agents. The mortality rate for infections in patients with beta thalassemia varies worldwide and it is related to epidemiology of each infection, the socio-economic level of each country and also the preventive and therapeutic strategies adopted. Herpesviridae represent a family of viruses that are pathogenic for many vertebrates. Several members of this large family cause longstanding infections that are characterized by periods of quiescence and reactivation(4), due to emotional stress, sun exposure, and menses(5). These viruses characteristically produce infections in the skin and squamous mucous membrane surfaces of the body. The two strains of HSV (serotype 1 and serotype 2) share many genomic and biological features. Initial HSV1 infection usually occurs in early childhood; primary infection is typically mild or asymptomatic. Despite cellular and humoral factors, HSV1 is able to migrate through sensory nerve fibers to the trigeminal ganglion and persists indefinitely in a dormant state in the neuronal nucleus. With severe impairment of cell-mediated immunity, reactivation infection may be severe or even fatal. HSV2 infection is found principally in genital squamous surfaces and is transmitted through intimate sexual contact. Primary and recurrent HSV2 lesions also heal completely in people with normal cellular immune function (6). It is suggested that HSV infection can aggravate the severity of anemia in patients with beta thalassemia by immune mechanisms. To our knowledge, data relevant to the seroprevalence of HSV infection in patients with thalassemia is limited. Due to dearth of information about seroprevalence of HSV infection in patients with beta thalassemia in Yazd, and for as much as we did not find any similar study, this study conducted to detect the prevalence of anti-HSV1,2 IgG antibodies in these patients.

## Materials and Methods

This study was approved by the Ethics Committee of Shahid Sadoughi University of Medical Sciences. This case control study included 45 particpants who were previously diagnosed as beta thalassemia major, according to their clinical and laboratory findings; and recruited from the specific diseases center of Shahid Sadoughi Hospital of Yazd, Iran. In addition, 45 healthy subjects were enrolled in the study. 

 Informed consent was obtained upon recruitment. Patients and controls were divided into three groups according to their age (age group 2-10 years, 11-20 years, and 21-30 years).

We undertook a serological study of HSV1,2 IgG antibodies among cases and controls by ELISA method. Four mLs of venous blood samples without anticoagulant were obtained from both groups. After about 15 minutes centrifuged at 3500 rpm for10 minutes, the serum was separated and frozen at (-20 ◦C) till used. HSV1,2 IgG antibodies were detected by ELISA technique (3rd generation ELISA, Abbott, Chicago, USA). Based on the cutoff value suggested by the manufacturer, the HSV1,2 IgG antibodies were considered positive at values greater than 10 IU/ml. 

**Statistical analysis**

Statistical analysis included frequency table and Fischer exact test . A p.value <0.05 was considered statistically significant. Statistical analyses were performed using SPSS 20.

## Results

**Figure1:**
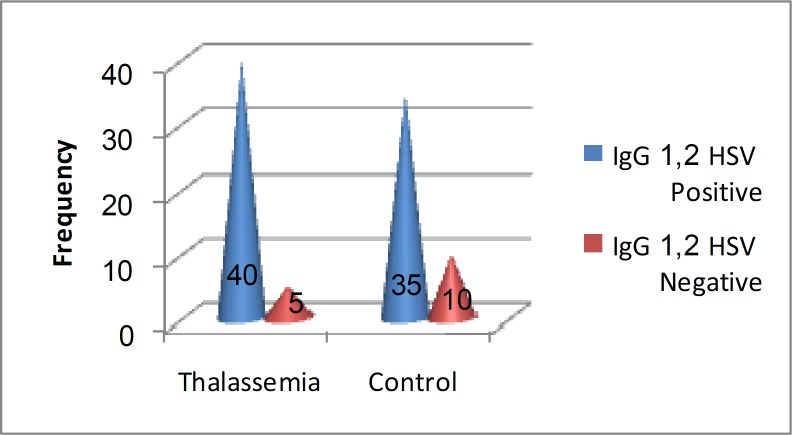
Shows the prevalence of HSV 1,2 IgG antibodies among two groups

There were 45 cases and 45 participants in the control group. In the case group, there were 20 males and 25 females and the mean age was 14.7±7.1 years. Twenty and twenty five participants were male and female respectively in the control group and the mean age was13.6±4.4 years. The prevalence of HSV 1,2 IgG antibodies in different age groups of patients and controls is shown in Table I. The prevalence of HSV 1,2 IgG antibodies were estimated 88.8% among patients with thalassemia and 77.7% in control group. Regarding p value=0.64, it showed no significant difference in these two groups (Figure1).

**Tabel I:** Reveals HSV1,2 antibodies status in cases and controls according to age

Total	HSV 1,2 antibodies Results		Age	GROUP
	Positive	Negative		
12	11	1	0-10	*Case*
23	22	1	11-20	
10	9	1	21-30	
45	42	3		Total
17	12	5	0-10	Control
28	23	5	11-20	
-	-	-	21-30	
45	35	10		Total

## Discussion

This study analyzes the prevalence of anti-HSV 1,2 antibodies in patients with thalassemia in a major tertiary care hospital located in Yazd. About 3% of the world’s populations (150 million people) carry β-thalassemia genes (7). In addition to anemia, these patients suffer from too many problems, including increased susceptibility to infections, which plays a major role in the patient's morbidity and mortality. Although much progress has been made, infectious diseases still represent a major challenge in the efforts for assuring prolonged survival in these patients. The prevalence of infection in multi transfused patients varies in different parts of the world and is directly related to the frequency in that population. To illustrate the increased susceptibility of these patients to infection, many researches have been carried out to investigate the possible changes of the immune function in patients with beta thalassemia; however no consistent defect in immune system had been documented. Different immunological defects are reported in some studies such as, decreased opsonization and white blood cells phagocytosis (8), increased serum immunoglobulin levels (9) disturbance in number and function of lymphocytes (10) and also changes of complement level (11). In addition, iron overload was supposed by some researcher as an important factor in immune disturbance (12). Nevertheless, the mentioned immune alteration may be a secondary immune defect rather than a primary one. On the other hand, infection with HTLV1 and HIV can lead to alterations in humeral immune system secondary to T-cell immune response depression (13) .The risk of transfusion-transmitted viral infection is well known in these patients. Hepatitis B Virus (HBV), Hepatitis C Virus (HCV), HIV and (HTLV-1) are dreaded consequences of transfusions, as these can result in long-term morbidity and mortality. HSV-1 and HSV-2 cause a similar set of primary and recurrent infections. These viruses produce acute and latent infections. In addition to causing cutaneous lesions, HSV-1 is the major infectious cause of corneal blindness in the United States; corneal epithelial disease is thought to be due to direct viral damage, while corneal stromal disease appears to be immune mediated. In addition, neonates and individuals with compromised cellular immunity may suffer disseminated herpes virus infections (14). It is suggested that autoimmune hemolytic anemia may occur in the course of some viral diseases such as herpes simplex virus. As a result, HSV infection can aggravate the severity of anemia in patients with beta thalassemia. To our knowledge, there is few data relevant to the seroprevalence of HSV infection in patients with thalassemia. In one study the prevalence of human herpes virus6 (HHV6) infection among pediatric patients with beta-thalassemia/HbE was investigated and the authors showed that the prevalence of HHV6 infection in these patients was very high (15). Another research demonstrated the human herpesvirus-6 as the etiology of hemophagocytic syndrome in a patient with thalassemia (16). However, we did not find any similar study that evaluate the seroprevalence of HSV1,2 antibodies in patients with thalassemia. Our results revealed no significant difference regarding past infection (IgG positive only) among the case and control groups.

## Conclusion

Although infectious diseases still represent a major challenge in patients with thalassemia, HSV1,2 past infection rate was not increased in these patients in our study. More investigation on more patients and in different parts of the country is required to clarify this matter.
